# Evaluating the Ability of Multi-Sensor Techniques to Capture Topographic Complexity

**DOI:** 10.3390/s21062105

**Published:** 2021-03-17

**Authors:** Hannah M. Cooper, Thad Wasklewicz, Zhen Zhu, William Lewis, Karley LeCompte, Madison Heffentrager, Rachel Smaby, Julian Brady, Robert Howard

**Affiliations:** 1 Department of Geography, Planning, and Environment, East Carolina University, Greenville, NC 27858, USA; wasklewiczt@ecu.edu (T.W.); lecomptek18@students.ecu.edu (K.L.); heffentragerm14@students.ecu.edu (M.H.); smabyr19@students.ecu.edu (R.S.); bradyj13@students.ecu.edu (J.B.); howardro@ecu.edu (R.H.); 2 College of Engineering and Technology, East Carolina University, Greenville, NC 27858, USA; zhuz@ecu.edu; 3 UAV Program, Pitt Community College, Winterville, NC 28590, USA; wlewis@email.pittcc.edu

**Keywords:** structure-from-motion, terrestrial laser scanning, lidar, OBIA, UAS, precision

## Abstract

This study provides an evaluation of multiple sensors by examining their precision and ability to capture topographic complexity. Five different small unmanned aerial systems (sUAS) were evaluated, each with a different camera, Global Navigation Satellite System (GNSS), and Inertial Measurement Unit (IMU). A lidar was also used on the largest sUAS and as a mobile scanning system. The quality of each of the seven platforms were compared to actual surface measurements gathered with real-time kinematic (RTK)-GNSS and terrestrial laser scanning. Rigorous field and photogrammetric assessment workflows were designed around a combination of structure-from-motion to align images, Monte Carlo simulations to calculate spatially variable error, object-based image analysis to create objects, and MC32-PM algorithm to calculate vertical differences between two dense point clouds. The precision of the sensors ranged 0.115 m (minimum of 0.11 m for MaRS with Sony A7iii camera and maximum of 0.225 m for Mavic2 Pro). In a heterogenous test location with varying slope and high terrain roughness, only three of the seven mobile platforms performed well (MaRS, Inspire 2, and Phantom 4 Pro). All mobile sensors performed better for the homogenous test location, but the sUAS lidar and mobile lidar contained the most noise. The findings presented herein provide insights into cost–benefit of purchasing various sUAS and sensors and their ability to capture high-definition topography.

## 1. Introduction

The ability to capture repeat, high-definition topography has rapidly evolved to allow users to quickly amass data at a variety of spatial and temporal scales [1,2]. Knowledge of short-term changes within a host of earth surface systems have been significantly advanced with the aid of high-definition topographic data [3,4,5], and these data provide further opportunities to examine human impacts on topography [6]. However, studies quantitatively assessing multiple widely available and newly evolving topographic data capturing approaches has been missing in the peer-reviewed literature. This is not to say examples do not exist [7,8,9,10]. However, these examples do not provide an evaluation of the wide range of sensors available at a single site in a manner that examines the precision of the sensors and the ability of the sensors to capture topographic complexity. Here, five different small unmanned aerial systems (sUAS) from Da-Jiang Innovations (DJI) each with a different camera were employed to fill-in this knowledge gap. A lidar (Sick LD-MRS for sUAS) was used on the largest sUAS. All sensors captured topography along a local engineered stream. The same airborne lidar affixed to the largest sUAS was also carried by hand on the ground to simulate a mobile scanning system mounted on a vehicle moving through the landscape. Traditionally, real-time kinematic global navigation satellite systems (RTK-GNSSs) surveying and terrestrial laser scanning (TLS) data were also collected in conjunction with the mobile sensing systems to examine spatial quality and representation of a surface [11,12]. RTK-GNSS and TLS datasets in the current research represent control data to examine the precision of the various mobile sensing platforms and their ability to capture the morphometry and topographic complexity in our field site. The findings presented herein provide insights into cost–benefit of purchasing various sUAS and sensors, precision of the sensors across a gradient of complex terrain, and their ability to capture high-definition topography, which is a fundamental boundary condition in numerical and physical modeling experiments [13] as well as change detection [13,14].

Much of the recent work examining the capability of sUAS sensors to capture complex topography has focused on validating these platforms against RTK-GNSS and total station surveying techniques as well as airborne laser scanning (ALS) and TLS [7,8,9,15]. RTK-GNSS, ALS, and TLS have often been used as reference data to compare sUAS sensors because they represent direct measurements of the topography [16] or greater accuracy in their measurements, particularly in the case of RTK-GNSS, total stations, and TLS surveying campaigns [17,18,19]. While a majority of the literature has shown good comparison of results with traditional surveying techniques, sUAS sensors were found to produce the higher quality and spatially more continuous topographic data than the ALS data when compared to RTK-GNSS data [15].

In general, there is a good agreeance between topographic data captured with TLS and sUAS sensors, but the literature does highlight some inconsistencies in topographic data captured with both TLS and sUAS sensors when compared with RTK-GNSS data [16]. This is particularly the case in settings with complex, heterogeneous terrain [7,8]. Some studies found TLS performed better in less complex topography and in areas with limited or no vegetation, but both TLS and sUAS sensors had difficulty in very densely vegetated settings [8]. sUAS sensors and TLS have also been noted to have higher discrepancies in sharp topographic transitions when compared with RTK-GNSS data [7].

These noted differences can be associated with a variety of user and instrumental errors. The experience of the authors suggests some of the differences may reflect field protocols/methods associated with the surveying campaigns [20]. In particular, the number, orientation, and location of positions used when sampling the topography with the TLS can reduce occlusions in the associated point cloud data [20] or the image geometry networks of the sUAS data capture [21].

Another important aspect noted with regard to quality of the TLS and sUAS sensor topographic data production is georeferencing [17]. Georeferencing accounted for up to 50% of the differences between the two data collection approaches, but site topographic disparities did not yield any significant differences [17]. Several studies have identified that remaining portions of the error not associated with georeferencing is often captured in multiple factors: flight paths, sensor location, and sensor parameters (i.e., camera calibration, image overlap, number of images, image geometry), geometry and number of GCPs, accurate measurement of GCPs, texture, lighting, weather conditions at the survey site, and image matching performance [8,17,20,22]. Another component adding to the differences in the sUAS and TLS might be the height the sUAS data are collected [9], as it has been shown that lower altitude flights (25–50 m AGL) are more accurate in the horizontal dimensions, but as a results of “doming” issues may have lower accuracy in the vertical dimension when compared with higher altitude flights (120–350 m AGL). The effect of flight patterns and heights should also be considered before flight planning to reduce systematic error in the image network e.g., [9].

Here, we expand on this existing literature and investigate multiple sUAS platforms and sensors using proven uncertainty measures [23,24,25], novel object-oriented image analyses techniques, and more traditional approaches to geomorphometric analyses. The goal is to assess which of the five sUAS platform performs best in complex terrain when compared against RTK-GNSS and TLS. The importance of this study is several fold: (1) to bring to bear multiple sUAS systems at one site at one time to assess performance of the various sensors for capturing accurate complex topographic data, (2) to inform future research in complex topography about the potential shortcomings in capturing accurate topography that is critical to many studies interested in environmental change detection, and (3) inform scientists from a variety of fields on what to expect from the various sensors that will aid in future planning and budgeting of research where topography is a critical underlying boundary condition to the study.

## 2. Materials and Methods

### 2.1. Study Area

**Figure 1:**
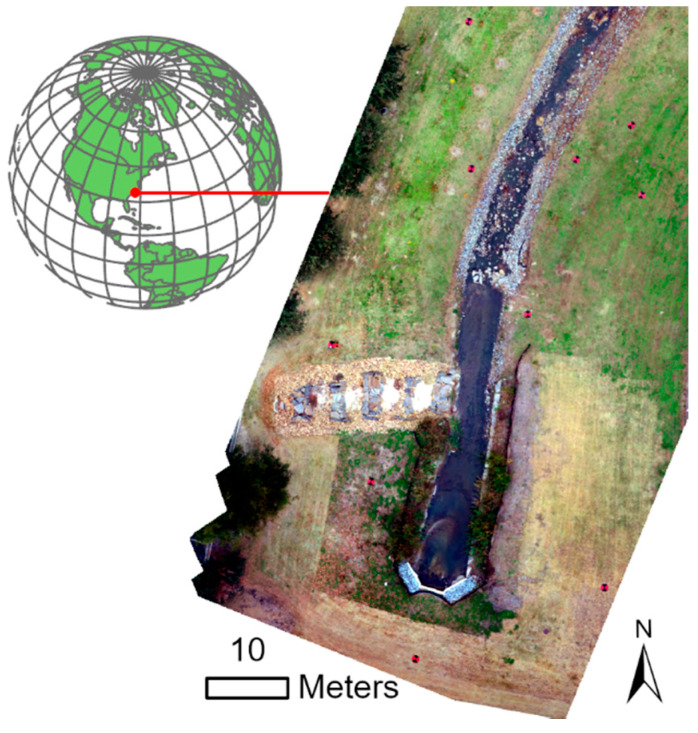
The study area of a channelized stream located in eastern North Carolina, USA.

The study area is a channelized stream located at approximately 35°36′45.63″ N and 77°22′08.46″ W in eastern North Carolina’s Coastal Plain physiographic region (Figure 1). The channelized stream redirects a natural stream in an artificially modified stream bed, which flows to the Tar River to help control flooding. Channelization deepened the stream channel, which increases the stream gradient. Reengineering included placing boulders and rocks along the channel in attempt to reduce future erosion. A freshly mowed lawn covers most of the upper slopes of channel. Elevations range 3.5–16.6 m above North American Vertical Datum of 1988 (NAVD 88) using Geoid 12 B. Slopes range from 0 to 89°, while terrain roughness (standard deviation of the slope) ranges from 0 to 10.

### 2.2. Field Data Collection

#### 2.2.1. RTK-GNSS Survey

RTK-GNSS measurements were used in this study to obtain the coordinates of ground control points (GCPs) to increase the absolute and relative accuracy of the reconstructed 3D point clouds. Prior to establishing GCPs, we created a benchmark using the Trimble Spectra Precision SP80 system with a reported high-precision static post-processed precision of 3 mm in the horizontal and 3.5 mm in the vertical (root mean square error (RMSE)) [26]. The +240 channels dual frequency SP80 receiver was used with full utilization of signals from all 6 GNSSs (GPS, GLONASS, BeiDou, Galileo, QZSS, and SBAS) while collecting data at 1-s intervals continuously for 8 h. The receiver’s raw file was then automatically adjusted with the National Oceanic Atmospheric Administration’s (NOAA) Online Positioning User Service (OPUS) solution using Trimble Business Center v5.10 with an overall benchmark RMSE of 0.015 m (Table 1).

A total of 19 GCPs were collected and consisted of fixed targets with a 0.9 m diameter high visibility black and orange right angle pattern with large numbers printed on rugged polyester. Each target’s perimeter was weighted with a 1.45 kg galvanized steel cable, and the center included a surveyor staff brass grommet for pinpoint accuracy in RTK-GNSS measures. The GCPs were distributed randomly by placing targets at breaks-in-slope to obtain measures that represent the complexity of the topography. Each target’s center was secured with survey nails driven flush to the ground. Three SP80 receivers were used in the survey, wherein one was set-up as a base station over the established benchmark. The other two SP80 receivers were used as rovers and placed over the center of each GCP at 180 fixed epochs using 1-s intervals averaged in RTK mode. During the survey, an additional 6 TLS targets were collected to register the TLS point cloud. The maximum standard deviation for all RTK-GNSS measures combined revealed 0.023 m in the horizontal (base overall error of 0.015 m + 0.008 m) and 0.028 m in the vertical (base overall error of 0.015 m + 0.013 m).

#### 2.2.2. TLS Survey

TLS data were used as the reference point cloud to compute distances between each of the 5 sUAS point clouds, the lidar affixed to the largest sUAS (unmanned lidar system or ULS), and mobile lidar system (MLS) point clouds. TLS data were collected using a Leica ScanStation P40 with a reported 3D position precision of 3 mm (1 standard deviation) at 50 m [27]. Twenty-one scan stations were set-up latterly across the channelized stream no more than 15 m apart to ensure suitable overlap. Registration and georeferencing was performed in Leica Cyclone (v. 9.x) using six 0.1524 m solid SECO sphere targets. The 21-point clouds were transformed using RTK-GNSS locations (precision of 0.023 m in the horizontal and 0.028 m in the vertical) associated with the center of each target. The registration or mean target distance error was 0.002 m. Thus, the overall error does not exceed 0.025 m in the horizontal (0.023 m + 0.002 m) and 0.03 m in the vertical (0.028 m + 0.002 m). Automatic classification of the points was performed in Agisoft Metashape Pro (v.1.6.2), where the algorithm misclassified some points as omission errors, and thus these points were manually classified back into the ground class. We note that the surface does not represent a true “bare earth” point cloud, as much of the surface was covered with freshly mowed grass.

#### 2.2.3. ULS and MLS-Based Surveys

A sensor packaged based on a compact lidar was reconfigured for this study in 2 setups: a hand-held ground mobile system, and a sUAS. The package includes: (1) SICK rugged multi-layer scanner (LD-MRS) Unmanned Aerial Vehicle (UAV) Lidar [28], (2) intelligent industrial cameras (IDS) Universal Serial Bus (USB) cameras, and (3) a NovAtel GNSS satellite receiver with a GNSS-inertial integrated Synchronous Position, Attitude and Navigation (SPAN) A1 navigator [29]. The receiver includes an ADIS16488 Inertial Measurement Unit (IMU) made by Analog Devices. The angular preciseness is reported as 0.012° for roll, 0.012° for pitch, and 0.074° for heading (one standard deviation) after post-processing [29]. In addition, a GoPro camera was used to record live videos during the process. The sensor package hardware remains the same in both setups. In the handheld mode, the sensor package is orientated such that the lidar is pointing forward, and a GNSS antenna is installed on an extension arm to point up. In the sUAS mode, the sensor package is attached rigidly to the sUAS body frame with a carbon fiber sensor mount. The antenna is installed on the top of the sUAS (DJI Matrice 600 Pro).

The SICK Lidar scans at 4 elevation angles simultaneously [28]. The field of view is approximately 110° horizontal and 3.2° vertical (evenly divided into 4 layers). The aperture size is approximately ±0.4° vertical and ±0.04° horizontal. The horizontal scans are 0.125° apart. The default scan rate is 12.5 Hz. A complete scan is associated with a single pose. It takes approximately 10 ms to complete 1 scan, and we are synchronizing to the middle of the scan. The discrepancy in timing is up to ±5 ms and ±2.5 ms on average for each point and is considered part of the error sources. The specification of this lidar includes a conservative estimation of ranging accuracy, with noise $\sigma_{R}\;\sim \;0.1\;m$ (quantization step 0.04 m) and systematic error $b_{R}\;\sim \;0.3\;m$. The systematic error could be calibrated in advance of the survey flight.

The sensors in this package are tightly integrated and precisely synchronized with each other. The lidar directly sends a hardware timing trigger signal to the GNSS receiver/navigator, and the IDS camera receives a timing trigger signal from it. The trigger signals can help establish synchronization at the millisecond level. Furthermore, to achieve higher accuracy in position and orientation measurements, we did not use the real-time solutions from the GNSS receiver/navigator to produce the point cloud. Instead, post-processed differential GPS/RTK-GNSS solution with respect to a nearby reference GNSS station in Greenville, NC, was used for positioning. Similarly, post-processed, tightly coupled RTK-GNSS-IMU integration solution was employed. Both were calculated through the NovAtel Waypoint and Inertial Explore software suite. The Inertial Explore software also provided us with post-processed kinematic positioning solution (the same as the NovAtel Waypoint software). The post-processed orientation and positioning accuracy was expected to be better than the real-time solution from the SPAN receiver. Since the post-processed orientation and positioning solution was sufficiently accurate, we did not need to provide any additional corrections to the point cloud. However, we did include laser calibration targets placed at surveyed locations in the raw 3D point cloud presented in this work. We used the 3D point cloud observed on these targets to validate the accuracy and precision of the over-all point cloud (±0.08 m, one standard deviation).

#### 2.2.4. sUAS-Based Surveys

**Figure 2:**
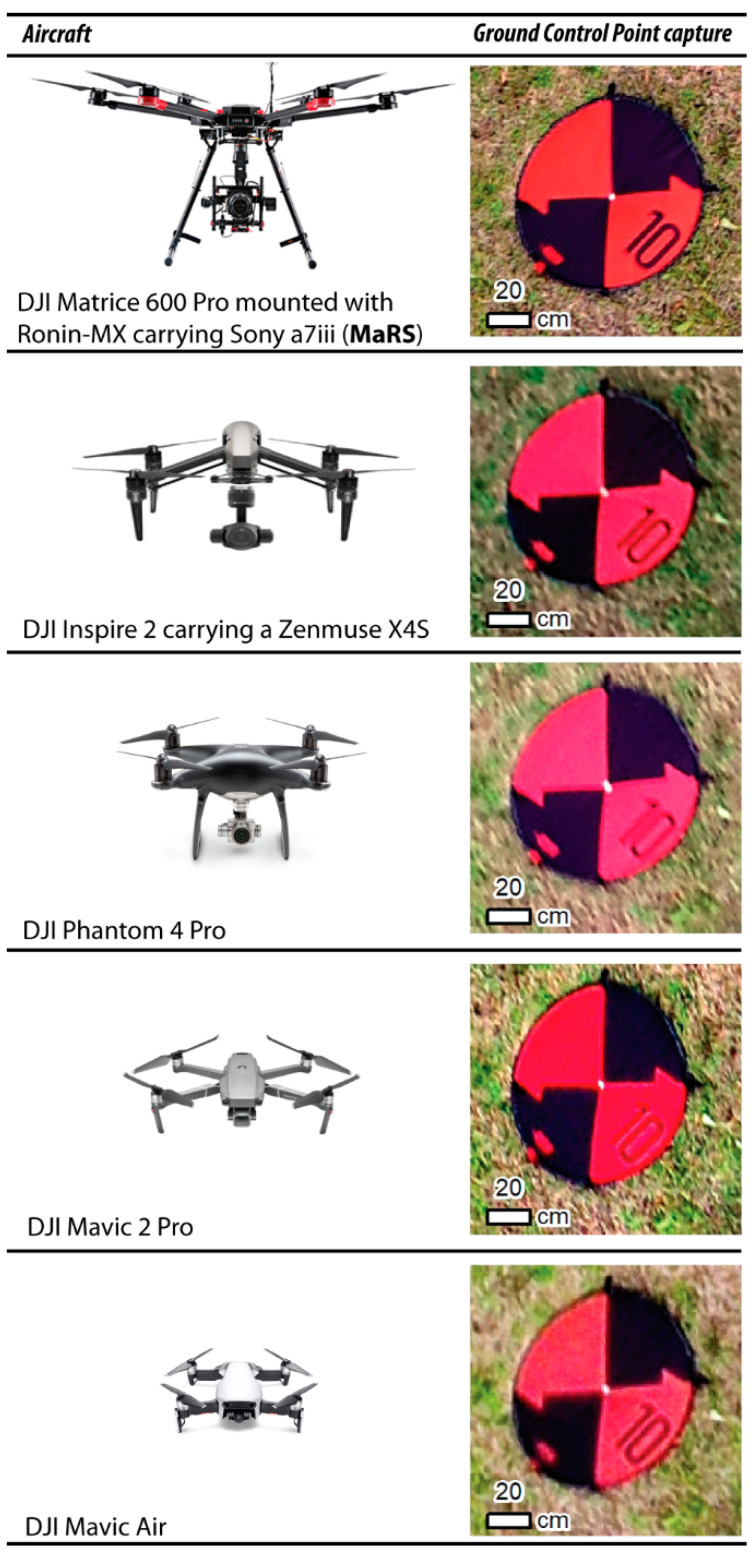
The five aircraft used in this study along with an example of each camera’s ability to capture a Ground Control Point (GCP). Aircraft image source: https://www.dji.com/ (accessed on 10 October 2020).

Two professional and three consumer-grade sUAS were evaluated for capturing topographic complexity (Figure 2). The first professional sUAS is a DJI Matrice 600 Pro mounted with the DJI Ronin-MX gimbal caring a Sony a7iii camera, which is referred to in this study as MaRS. The second is a DJI Inspire 2 carrying a Zenmuse X4S camera. The three consumer-grade sUAS include a DJI Phantom 4 Pro, DJI Mavic 2 Pro, and DJI Mavic Air Mini. The specifications of each platform and their respective camera are shown in Table 2.

Atmospheric conditions can impact the quality of the images as well as flight safety. Prior to and during flight operations, the notices to airmen (NOTAMs; identifies where a pilot can fly) information was obtained in addition to aviation weather report (METAR) and terminal aerodrome forecast (TAF) from the National Weather Service Aviation Weather Center at www.aviationweather.gov. All flights were conducted by Federal Aviation Administration Part 107 certified remote pilots on 27 November 2019 with southwest winds of 5.4–7.2 m/s, visibility > 9.66 km, sky cover broken at 1800 m above ground level, and temperatures between 17.2 and 22.2 °C. Since multiple flights were conducted in 1 day, a uniformly cloudy day was preferred in an attempt to decrease shadows, which would reduce the quality of the 3D reconstruction.

DJI Ground Station GS Pro (v.2.0) was used to plan all flight missions because of its compatibility with each aircraft and pilot control over several flight parameters. Our goal was to set all flight parameters equal for comparison purposes; however, this was not practical due to unique camera and aircraft features (see Table 3 for detailed flight parameters used in this study). For example, due to the unique configuration of MaRS (e.g., aircraft’s size and weight), the flight planning software restricts the aircraft to hover and capture at a point. This makes the velocity different from equal distance interval; therefore, any photos with blur will be quantified and eliminated in this study before image processing for fair comparison. Two flights were carried out by each of the 5 aircraft (a total of 10 flights). The Shooting angle of perpendicular to main path was chosen, meaning the sUAS faced the same direction perpendicular to the main flight lines. The main flight lines were chosen using a single parallel pattern (zig-zag scheme) flown at 30° and then 120°. These 2 course angles will then be combined into a crosshatch pattern. We also chose a −70-degree gimbal pitch angle because when combined with a double hatch pattern in the flight planning missions, image geometry will be increased, which has shown to reduce systematic error. All cameras were set to auto for shutter speed and image sensor sensitivity (ISO), and focus was set to infinity.

### 2.3. Data Processing and Analysis

**Figure 3:**
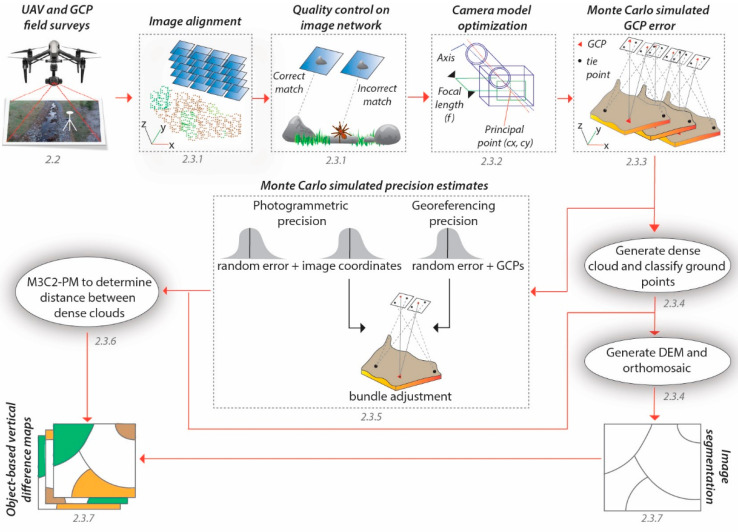
Illustration of the workflow used in this study. The subsections are indicated for clarity (e.g., 2.2 relates to Section 2.2).

A workflow was created in this study by combining the methods of several proven data processing and analysis techniques into one. Once the images and GCPs were collected in the field, structure-from-motion (SfM) techniques were used in the workflow to align the images by identifying and matching common features on overlapping images into tie points (Figure 3). A quality control assessment was performed on the image network by checking for errors in the potential mismatching of tie points [23]. Different camera model parameters were then tested to evaluate their performance [23]. Monte Carlo simulations were generated to randomly select different GCPs as control so that the xyz error of each georeferenced image network could be assessed [23,24]. The refined image network was then used in the following 2 ways. First, to generate dense clouds that were classified as ground points so that a Digital Elevation Model (DEM) and orthomosaic could be created to generate image objects. Second, a Monte Carlo approach was used once again, but this time to simulate photogrammetric and georeferencing precision estimates that were incorporated into the M3C2-PM algorithm to detect any significant changes between two-point clouds [24,25,30]. Finally, the mean of the vertical differences between two-point clouds were assigned to an object when more than 1 point fell within that object. The results were object-based vertical difference maps that detect any changes that are statistically significant at the 95% confidence level.

#### 2.3.1. sUAS Image Alignment

After acquiring overlapping images of the terrain from different flight pattern angles, we used the automatic image quality feature in Agisoft Metashape Pro (v.1.6.2) to exclude any poorly focused images, which can have an adverse effect on the image alignment. While Agisoft Metashape Pro recommends disabling images with a quality value of less than 0.5 units, we chose a more conservative value by removing any images with a quality value less than 0.8 units (note: no more than 4 images were removed from each network, meaning the great majority of all images captured were well focused). SfM was then used to reconstruct 3D geometry by identifying and matching common features on overlapping images into tie points. Images were aligned using Agisoft Metashape Pro by executing the “align images” function with the following parameters: “high” accuracy, generic pre-selection, and 50,000 key points (the maximum number of features) and 5000 tie points (the maximum number of matching common features). A bundle adjustment was then carried out using camera parameters of focal length (*f*), principal point (*cx*, *cy*), and radial distortion (*k*1, *k*2, *k*3). This least-squares global optimization approach reduces image residuals by adjusting camera parameters, camera orientations, and the 3D point positions [23,31]. The output from this procedure results in a more reliable aligned image network based on the estimated camera positions from the imagery alone and a resulting sparse cloud. A quality control assessment was then performed on this initial network by checking for errors associated with the potential mismatching of tie points or images that may need removal (R v.4.0.2 was used to visualize the results). Image networks with the lowest camera reprojection errors were attained, which is the image RMSE in pixels or square root of the averaged squared distances between the projected reconstructed valid tie points and their corresponding original projections detected on the photos. Once an image network was checked for errors due to the potential mismatching of tie points or images, the GCPs were slowly added into the network.

GCPs were identified as markers on the images and added to the network by first performing a “free” bundle adjustment where no GCPs are set as control [23]. This allowed for the quality of the GCP image observations to be captured in pixels before linking the GCP ground coordinates to the network. Georeferencing to an established coordinate system was then achieved by matching the identified GCPs with their 3D ground coordinates accompanied by another bundle adjustment using all GCPs as control points. Networks with the lowest marker RMSE of the distances between the estimated positions of each marker and the corresponding independent source of higher accuracy (GCP 3D coordinate) were obtained and the results were visualized using R. A total of 5 image networks were produced and ready to be tested with different camera models for the best results.

#### 2.3.2. sUAS Camera Model Optimization

Two different camera model parameter sets were assessed for each of the 5 networks by evaluating their performance on the basis of which provided the smallest RMSE (totals to 10 evaluations because there were 5 networks each tested with 2 camera models (5 networks × 2 camera models = 10)). Camera model parameter sets tested in this study were models B and C, as shown in Table 4. These models were found to be conservative when compared to other model parameter sets tested in another study [23]. A conservative model was preferred because it improves the overall network performance by decreasing the RMSE on both check and quality control points. Therefore, we split the GCPs, wherein half was designated as control and the other half was designated as quality. Following the novel study by [23], we adopted their Monte Carlo approach used in Agisoft Metashape Pro along with the sfm_georef v.3.0 software by [25] to estimate error using 50 different random selections of GCPs as control. Since the principle of Monte Carlo simulation follows the law of large numbers theorem by averaging the results over many trial runs, it provides a more reliable result of the expected error. The results supported the use of camera Model B for all networks except one, the Mavic Air Mini. With the camera models fixed, another bundle adjustment was carried out using all GCPs as control to capture the final parameter values used to assess the overall quality of each network or GCP error (Table 5).

#### 2.3.3. sUAS GCP Error Assessment

The image alignment and camera model optimization steps above were repeated, but this time the errors and camera model for each aircraft were included in the bundle adjustment, as shown in Table 5. Again, we followed [23] using their Monte Carlo approach in Agisoft Metashape Pro to calculate the control and quality GCP RMSE using 50 different random selections of GCPs as control. The quality of each survey was then visualized in R.

#### 2.3.4. sUAS Dense Point Cloud Generation

With high-quality networks generated from each SfM-based survey, we generated a dense point cloud for each and classified as ground points. In this process, all GCPs were marked as control and the errors and camera model for each aircraft were included in the bundle adjustment. Dense point clouds were generated using the settings of “high” quality and “aggressive” depth filtering. Next, the automatic classification procedure in Agisoft Metashape Pro was used, which first divides the dense point cloud into cells where the lowest point of each cell is triangulated into a terrain model. A candidate point is classified as ground if it is within a certain distance of an angle between the terrain model and a classified ground point. We tested different parameters on the basis of trial and error for cell size, max distance (distance between candidate ground point and terrain model), and max angle (angle between the terrain model and line that connects a classified ground point with a candidate ground point). A visual inspection of the automatic classification of ground points indicated another round of filtering was needed, which was accomplished through manual classification, followed by another visual inspection of the results. Results were 3D point clouds of classified ground returns ready to have their xyz spatially variable precision estimates added.

#### 2.3.5. sUAS Spatially Variable 3D Precision Estimates

We implement the novel Monte Carlo approach by [24] to derive 3D precision (1 standard deviation) maps containing the variation of both photogrammetric and georeferencing uncertainties. The Python script associated with [24] was edited for use in Agisoft Metashape Pro (v.1.6.2) for this study. In the automated analysis, image networks were created without error before random variables were sampled proportional to normal distributions using the RMSE of the original image residuals (it is assumed that the RMSE is equivalent to 1 standard deviation). The random errors were then added to the observations and control measurements followed by a bundle adjustment. The sampling procedure was repeated a total of 500 cases for each image network (5 image networks × 500 cases = 2500). The sfm_georef software [25] was then used to compile the Monte Carlo outputs into xyz precision estimates for each spare tie point. CloudCompare v.2.11 (cloudcompare.org) was then used to interpolate the xyz point precisions onto their individual grids using the 6 nearest neighbors and median to ensure all dense points had a precision value. The results were a total of 5 dense clouds with associated 3D precision estimates suitable for measuring any significant change from the TLS dense cloud.

#### 2.3.6. Vertical Difference Estimations Using M3C2-PM and M3C2

The SfM-based dense clouds were initially compared with the TLS dense cloud. The M3C2 algorithm [30] was used in CloudCompare to calculate distances between them because each dense cloud contains a uniquely defined structure. The M3C2 algorithm works by estimating and orienting surface normals at a scale of the local surface roughness before measuring the mean surface change along the normal direction [30]. The main parameters to adjust are core points (a sub-sampled version of the TLS dense cloud), normal scale (the diameter of a local neighborhood around each TLS core point used to compute a normal), projection scale (the diameter of the cylinder used to define the two sub-clouds to compute distance along the normal direction), and max depth (the cylinder height). In this study, the core points were subsampled to a value of 0.10, the normals were set to multiscale so that the scale where the cloud was most planar was selected (0.25–5 with increments of 0.25), and the max depth was set to 2. The initial 3D differences calculated by the M3C2 algorithm along with the 3D precision maps were then input into the M3C2-PM algorithm [23] to process confidence bounds and significant changes in the SfM dense clouds. For the ULS and MLS datasets, significant changes were calculated using in the M3C2 algorithm using the same parameters as above (core points = 0.10, normals = 0.25–5 with increments of 0.25, and max depth = 2). The results were a total of 7 dense clouds with their respective statistically significant vertical differences from the TLS surface.

#### 2.3.7. Object-Based Image Analysis (OBIA) Vertical Difference Mapping

The vertical differences between 2 point clouds can be structured as a grid with a regular shape and size or objects with varying shapes and sizes. Object-based image analysis (OBIA) offers the opportunity to match the differences between 2 point clouds to relatively homogenous objects rather than a grid cell in which the topography might be heterogenous in structure. OBIA has been used to classify topography from SRTM DEMs [32], detect landslides from airborne Lidar DEMs [33], classify undersea topography using SRTM30_PLUS DEMs [34], classify TLS point clouds [35], detect change for landslide monitoring [36], and correct airborne lidar point clouds to generate object-based DEMs [37]. While the application of OBIA in sUAS SfM-derived DEMs has gained attention [38,39], an object-based approach to detecting change in SfM-based dense clouds is missing. In this study, OBIA is used to map the vertical differences between SfM-based dense clouds and TLS, ULS and TLS, and MLS and TLS. We expect that an object is more representative of a topographic feature than an individual grid cell that is within that region.

OBIA was performed using the TLS point cloud and sUAS imagery that had the highest quality, which was the MaRS imagery (>0.92 units). After completing the sUAS-SfM data processing workflow in earlier steps, we created a 6 mm horizontal resolution orthomosaic from the MaRS imagery in Agisoft Metashape Pro. We then generated objects using the multiresolution segmentation algorithm in eCognition Developer 9.5.1 [40]. The algorithm first segments individual pixels of an image before merging neighboring segments together until a heterogeneity threshold is reached [40]. The heterogeneity threshold is determined by the following parameters: scale (defines the maximum standard deviation of the homogeneity criteria based on the weighted layers), color (digital value of a pixel), shape (defines the textural homogeneity of the image objects by totaling smoothness and compactness), smoothness (optimizes objects by how similar their boarders are to a square), and compactness (optimizes objects by how similar pixels clustered in an object are to a circle). The scale determines the size of objects where a smaller scale value produces smaller homogeneous objects, and a larger scale value produces larger heterogenous objects. To help identify an optimal scale for defining topographic features, we tested 4 segmentations using scale parameters ranging from 25 to 100 at an interval of 25. Two pairs of criteria were weighted to a value of 1 to define the relative homogeneity for the image objects. The first criterion color/shape was set to 0.8/0.2 for all layers so that spectral information was weighted most heavily for segmentation. The second criterion smoothness/compactness was set to 0.5/0.5 for all layers so that compact and non-compact segments were favored equally. After segmentation, the statistically significant point cloud outputs from the M3C2-PM and M3C2 procedures were then averaged for each object when more than 1 point falls within an object using ArcGIS Pro version 2.2 (http://www.esri.com/) (10 October 2020) Data Management tools. The results were object-based vertical differences significant at the 95% confidence level maps, 1 for each of the 7 datasets tested in this study.

## 3. Results

### 3.1. Image Network Quality Control Assessment

**Figure 4:**
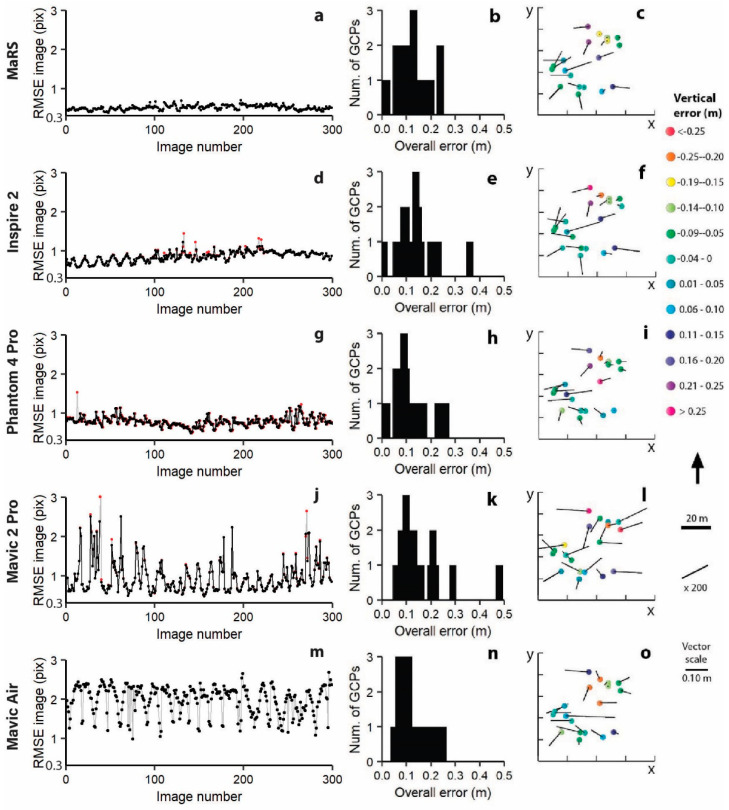
Assessment of network quality control for MaRS (**a**–**c**), Inspire 2 (**d**–**f**), Phantom 4 Pro (**g**–**i**), Mavic 2 Pro (**j**–**l**), and Mavic Air (**m**–**o**). (**a**,**d**,**g**,**j**,**m**) Root mean square error (RMSE) tie point image residuals in pixels for each image in each of the five networks where red indicates before image removal and black indicates after image removal. GCPs included as control in the bundle adjustment with a distribution of their overall error (xyz) (**b**,**e**,**h**,**k**,**n**), and 3D errors and GCPs where the vectors are the horizontal error magnified by 200 (**c**,**f**,**i**,**l**,**o**). The image root mean square error (RMSE) is the square root of the averaged squared distances between the projected reconstructed valid tie points and their corresponding original projections detected on the photos.

An initial visual examination of each aircraft’s sparse cloud of photogrammetric tie points did not permit a useful means of determining outliers in the estimated positions. Therefore, the RMSE between the projected reconstructed tie points and their corresponding original projections detected on the photos were calculated for each photo in each aircraft’s network (Figure 4a,d,g,j,m) following the work of [26]. The image networks for MaRS and Mavic Air did not show any photos with anomalously high image residuals (Figure 4a,m), and thus no photos were removed from these networks. However, the Inspire 2 (four photos), Phantom 4 Pro (one photo), and Mavic 2 Pro (three photos) had photos with anonymously high RMSE magnitudes, and thus these photos were removed from further processing of each of these networks (Figure 4d,g,j). We chose to remove no more than 30% of all tie points spread throughout each of the five image networks. This resulted in a threshold of >0.5 pixels for all image networks with one exception, all tie points that had image residuals >1.5 pixels were removed from the Mavic Air network due to the large range of RMSE values (Figure 4m). The removal of some of these tie points reduced the overall image RMSE from 0.51 to 0.50 pixels for MaRS, 0.85 to 0.79 pixels for Inspire, 0.78 to 0.75 pixels for Phantom 4 Pro, 0.97 to 0.82 pixels for Mavic 2 Pro, and 2.03 to 1.95 pixels for Mavic Air.

The overall image RMSE was then set for tie point “accuracy” (this is really the error), and the GCP field precision was set for marker “accuracy” before including all 19 GCPs as control in each bundle adjustment. The resulting overall errors (xyz) showed some discrepancies exceeding 0.3 m for Inspire 2 (Figure 4e) and Mavic 2 Pro (Figure 4k) when compared to the other three networks (Figure 4b,h,n). However, these GCPs were not considered as outliers, and all GCPs were used for further processing of each of the five networks. Overall, MaRS produced the most reliable image network with the smallest errors (Figure 4).

### 3.2. Ground Control Assessment

**Figure 5:**
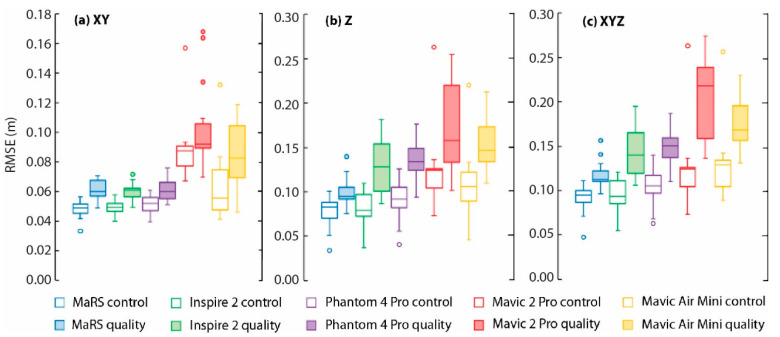
RMSE on control and quality points for Monte Carlo analysis on each sUAS-structure-from-motion (SfM) aircraft. A total of 50 self-calibrating bundle adjustments were carried out with a marker precision of 0.03 m and different random selections of 10 GCPs used as control points and 9 GCPs used as quality points. The center line in each box represents the median RMSE, and the box extends between the 25th and 75th percentiles. The whiskers represent the full range of the results, while the circles represent any outliers. In the figure, (**a**) xy is the horizontal errors, (**b**) z is the vertical errors, and (**c**) xyz is the horizontal and vertical errors combined.

As expected, the random selections of GCPs set as control have consistently lower errors than when compared to those GCPs randomly selected as quality (Figure 5). This is because the GCPs set as control contain bias, as they were used to georeference each point cloud at each simulation. Additionally, the higher errors of the GCPs randomly selected as quality indicates that the chosen parameters were effective at not overfitting the control points. For the xy positional errors, MaRS, Inspire 2, and Phantom 4 Pro all provided satisfactory low errors on the random GCPs set as quality (median RMSE ranges 0.06–0.061 m). However, MaRS provided a noticeably lower z positional error on randomly selected GCPs set as quality (median RMSE = 0.099 m) when compared to all other aircraft (median RMSE = 0.125 m for Inspire 2, 0.13 m for Phantom 4 Pro, 0.16 m for Mavic 2 Pro, and 0.14 m for Mavic Air). In reviewing the results of the xyz positional errors combined, we found that MaRS performed the best with a median RMSE of 0.11 m, while Mavic 2 Pro performed the worst with a median RMSE of 0.225 m.

### 3.3. Maps of Object-Based Vertical Differences Significant at the 95% Confidence Level

**Figure 6:**
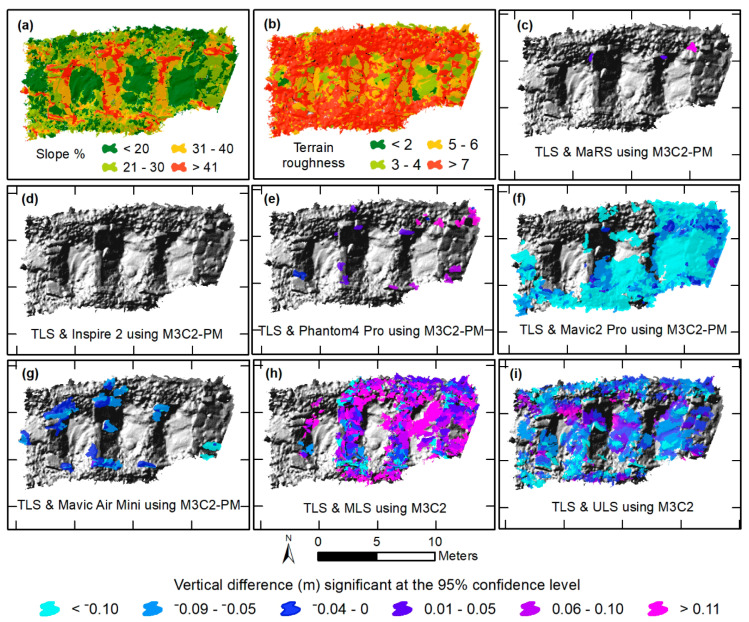
Object-based mean vertical differences of the original point clouds significant at the 95% confidence level between (1) terrestrial laser scanning (TLS) and SfM-based surveys using the M3C2-PM algorithm (**c**–**g**), and (2) TLS and mobile lidar system (MLS) (**h**), and TLS and unmanned lidar system (ULS) (**i**) using the M3C2 algorithm, all in meters. Object-based slope was determined using the TLS (**a**), and terrain roughness was determined using the standard deviation of the slope derived from the TLS (**b**). All objects are overlying a hill shade image.

**Figure 7:**
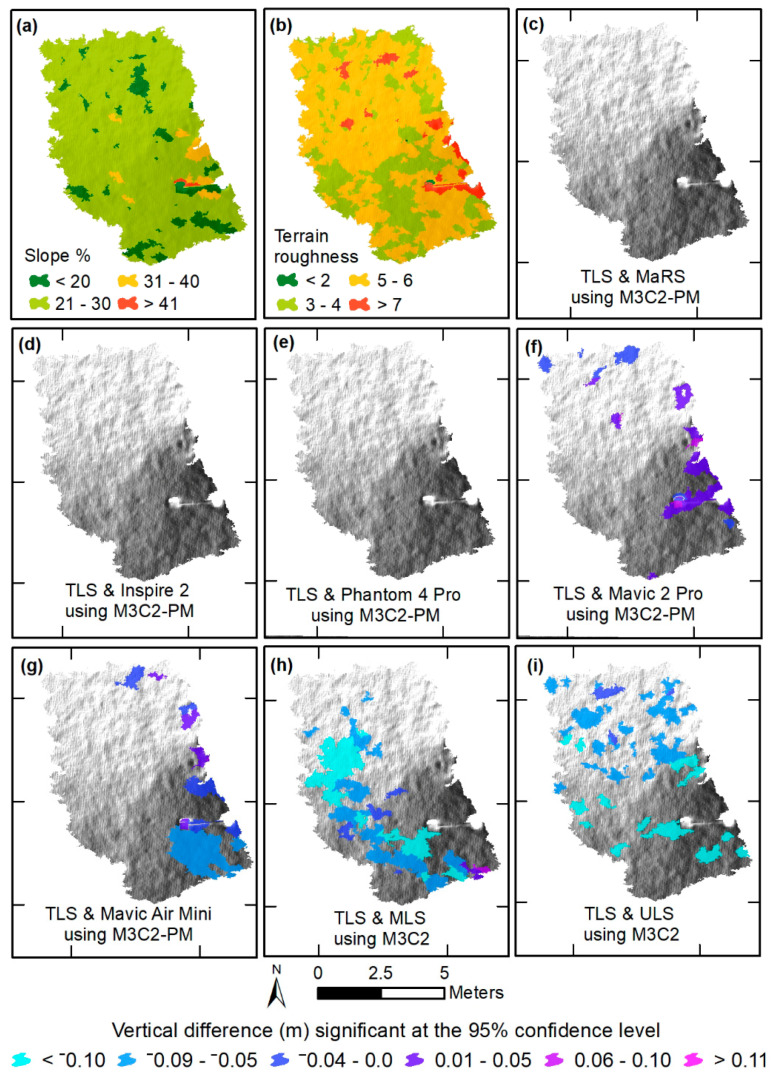
Object-based mean vertical differences of the original point clouds significant at the 95% confidence level between (1) TLS and SfM-based surveys using the M3C2-PM algorithm (**c**–**g**), and (2) TLS and MLS (**h**), and TLS and ULS (**i**) using the M3C2 algorithm, all in meters. Object-based slope was determined using the TLS (**a**), and terrain roughness was determined using the standard deviation of the slope derived from the TLS (**b**). All objects are overlying a hill shade image.

**Figure 8:**
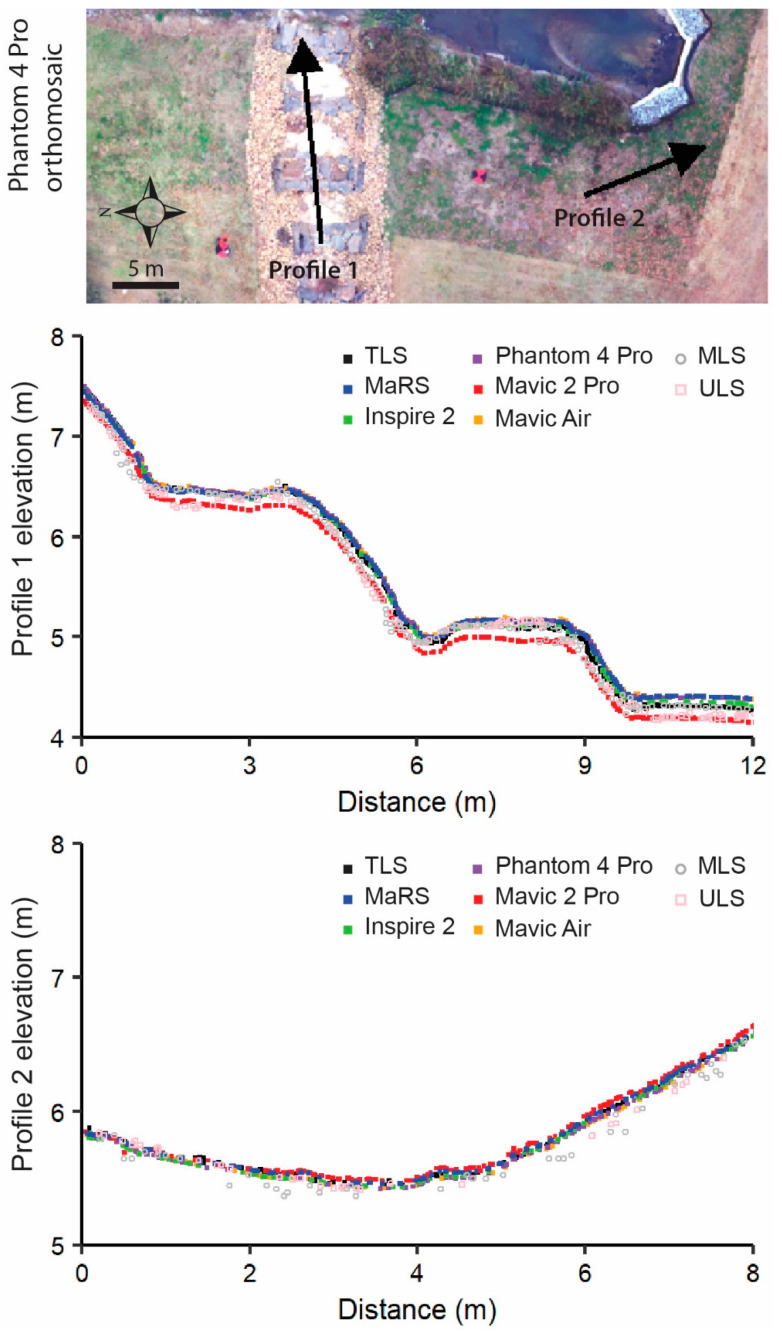
Vertical profiles of all dense clouds classified as ground where **profile 1** is rough terrain and **profile 2** is soft terrain.

The object-based vertical difference maps were derived by applying the point cloud outputs that were significant at the 95% confidence level from the M3C2-PM (Figure 6c–g and Figure 7c–g) and M3C2 (Figure 6h,i and Figure 7h,i) procedures to the object-based scale 50 data. Two locations were chosen on the basis of characteristics of slope and terrain roughness to demonstrate the effectiveness of each survey dataset’s ability to measure topography. The first is a rocky staircase (designed to convey impervious surface runoff and reduce flow velocity) with varying percentages of slope and a high amount of terrain roughness (Figure 6a,b), while the second location has a more uniform slope and low terrain roughness (Figure 7a,b). At the rocky staircase location, we found that MaRS, Inspire 2, and Phantom 4 Pro preformed best, as there were little or no areas that exceeded vertical differences at 95% confidence level (Figure 6c–e). This is likely a result of a combination of each aircraft’s global shutter camera, a consistent network of tie points that was less likely to mismatch rock features, and reduced uncertainty in the underlaying data. Additionally, the ULS and MLS datasets had a good majority of area that exceeded the 95% confidence level (Figure 6h,i). Observations indicate the higher degree of uncertainty is associated with noise and laser scatter among the rocks. While all datasets performed better for the second location of uniform slope and low terrain roughness covered in freshly mowed grass, the ULS and MLS still contained noise and a more extensive area that exceeded the 95% confidence level (Figure 7). The ULS and MLS noise are also evident in the vertical profiles for both locations (Figure 8).

## 4. Discussion

Our investigation of multiple sUAS systems at one site at one time using proven uncertainty measures [23,24] and object-oriented image analyses techniques [40] provides insights into future planning and budgeting of research where fine topographic data are critical. Sensor quality issues resulted from several factors such as sensor size, shutter speed, terrain roughness, and land cover. Only three of the seven sensors tested in this study were deemed suitable for accurate topographic mapping. Our findings, discussed in detail below, led us to posit that only one sUAS had the best tradeoffs between sensor quality, cost, portability, and functionality in capturing complex topography in an accurate manner deemed essential for environmental change detection, geomorphological and/or mapping, engineering geology problems, and environmental management designs.

### 4.1. Identifying the Best Sensor

The best sensor used in this study was the SonyA7iii (MaRS aircraft) because it had the largest sensor size, finest resolution of 24 MP (24 million pixels are recorded in a single image), wide range in global shutter speed for reducing motion blur, and a wide range of values for getting the proper exposure (Table 2). A larger sensor is better because it allows more photosites to capture light that is translated into more pixels, thus producing better images with higher quality resolution. The advantage of a high-resolution camera is that there are more pixels to work with when defining an object on the Earth’s surface. This is helpful to the SfM process when accurately matching tie points of corners and lines of features on the ground, as demonstrated by the Sony A7iii sensor (MaRS aircraft) having the strongest image quality network with the lowest RMSE tie point image residuals (see Figure 4a). In addition to the resolution of the sensor, the Sony A7iii camera has the largest range in global shutter speed. A sensor that deploys a global shutter is preferred because the sensor scans the entire area of an image simultaneously, allowing less time for motion blurred images. The Sony A7iii’s large sensor can also produce images with the right amount of exposure, or amount of light per unit area reaching the sensor. Getting proper exposure requires a delicate balance of lens aperture, shutter speed, and scene illuminance or ISO, and the Sony A7iii has the largest range for each when compared to all other cameras (Table 2). A strong image network of properly exposed images will only improve the orthomosaic and thus OBIA results because the heterogenous threshold is based on color and shape when defining image objects.

### 4.2. Performance of Various Sensors in Complex Terrain

The performance of the sensors on areas with different slopes and terrain roughness were tested because sensor performance can be influenced by the terrain being measured [8]. All sensors worked best for slopes less than 40% (or <22°) with a low terrain roughness (≤6) (note: terrain roughness is the standard deviation of the slope). Areas with high terrain roughness (>6) proved most challenging for camera sensors with rolling shutter speed (Mavic 2 Pro, Mavic Air) and the ULS and MLS. The disadvantage of a rolling shutter is that the sensor takes time to scan the entire area of an image sequentially from the top to bottom causing motion blur in the images, which is detrimental to many of the SfM algorithms used to generate point clouds. However, we did disable any images with a quality <0.8 units from image processing. While lidar’s advantage over SfM techniques is the ability of the laser to penetrate vegetation to capture ground measures [8], we found that the laser easily scatters among rocky terrain creating noise in the data. Camera sensor resolutions higher than 20 MP with global shutter speed (MaRS, Inspire 2, Phantom 4 Pro) performed well in both the heterogeneous and homogeneous terrain examined in this experiment.

While this study found that all sensors performed well for an area with a low terrain roughness (defined by ≤6 standard deviations of the slope), it was noted that the land cover was mowed lawn. In another study, sensor performance was poor for an area of homogenous terrain because the sensor was capturing reflectance from a white sandy beach [8]. The homogenous spectral signatures of a white sandy beach captured during sunny skies complicate the SfM process from detecting variation needed to match grain features of corners and lines associated with color gradients in the imagery.

A uniform cloudy day helped reduce the impacts from shadows during the collection of the imagery and to decrease reflectance issues in sand locations shown in the imagery derived from MaRS (e.g., see Figure 8) and Inspire 2. The Phantom 4 Pro, Mavic 2 Pro, and Mavic Air imagery in comparison all had poor exposure for the sandy pools (e.g., see Phantom 4 Pro imagery in Figure 8). The only difference between the Inspire 2 and Phantom 4 Pro sensors is the ISO range, where the Phantom 4 Pro ISO speed is about half that of Inspire 2. To properly capture exposed images of the sandy pools during our uniform cloudy day, we needed the minimum sensor requirements of Inspire 2 (see Table 2). However, there are other factors to consider that may affect the ability of the sensors to capture the shape of the terrain.

**Figure 9:**
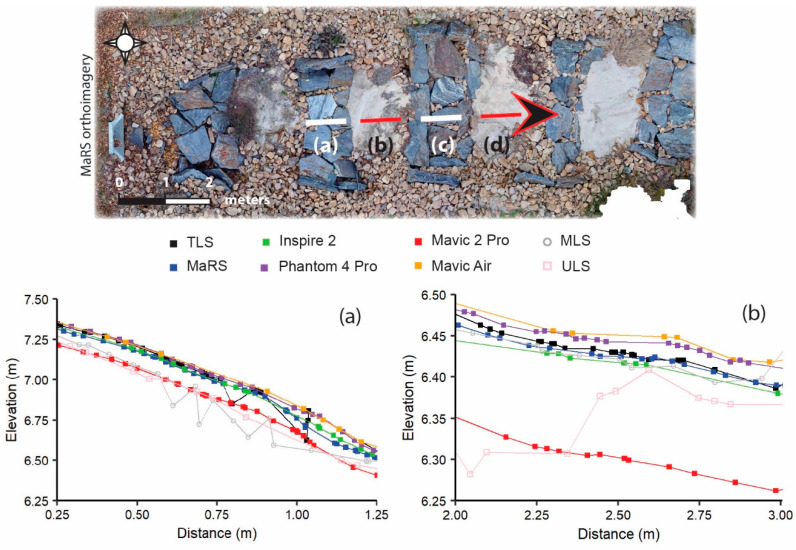

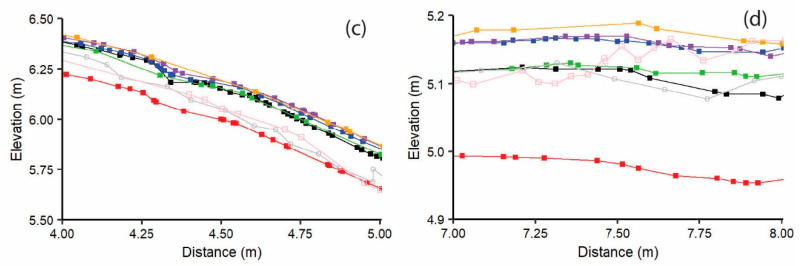
Smaller segments of vertical profiles of all dense clouds classified as ground for the rough terrain where segments (**a**,**c**) are rocky steps and segments (**b**,**d**) are pools of sand. All points that intersected the profile line were extracted from each point cloud. The lines represent connection of adjacent points.

The vertical profile for the rough terrain (profile 1 in Figure 8) is shown in detail by taking smaller 1 m segments of the rock steps (Figure 9a,c) and sand pools (Figure 9b,d). The Mavic Air captured the shape of the rock steps better than the Mavic 2 Pro, despite noticeable differences in lens aperture, shutter speed, ISO range, and resolution between the two sensors (see Table 2). This likely reflects differences in a combination of Mavic 2 Pro’s wide-angle lens (28 mm), manufacturer pre-processing conversion of raw imagery data, and land cover, but this supposition would require further testing. While the MaRS data closely follow the TLS for the sand pool in Figure 9b, there was a positive bias in the data when examining the second sand pool profile in Figure 9d. The MaRS’ wider lens (28 mm) may have caused some optical distortion in the raw images, in addition to the image geometry used in this study. The Inspire 2 provided mixed results on the flatter, sandy surfaces (Figure 9b,d), underestimating the surface in one location in comparison to the TLS (Figure 9b) and more closely approximating the shape of the surface compared to MaRS (Figure 9d). The Phantom 4 Pro does a relatively good job of representing the morphometry, but consistently overestimates the height (Figure 9).

### 4.3. Insights into Cost–Benefit of Purchasing Various sUAS and Sensors

There are also tradeoffs between sensor quality, cost, portability, functionality, and maintenance. While MaRS has the highest quality sensor, it is more expensive than Inspire 2 (second most expensive), Phantom 4 Pro (third most expensive), Mavic 2 Pro (fourth most expensive), and Mavic Air (least expensive). In addition, MaRS is not as portable as the other aircraft because of its weight and large size (1.7-m diameter with propellers and frame arms unfolded). However, the functionality of MaRS makes it attractive to the authors, as the aircraft has a higher pay load so that heavier sensors can be carried such as the DJI Ronin-Mx gimbal carrying the Sony A7iii camera and the SICK lidar system. Another con is that MaRS requires more maintenance and experience because there are many individual parts that work together such as the aircraft, gimbal, GNSS, IMU, and the sensor that all require individual attention. While the next best sensor to the MaRS Sony A7iii is the Inspire 2 Zenmuse X4S camera, the Inspire 2 aircraft itself is more portable, requires less experience, and is easier to maintain. Given all these pros and cons, the authors prefer MaRS or Inspire 2 for measuring complex topography. However, we agree with another study [41] that the Phantom 4 Pro provides the best tradeoff between sensor quality, cost, portability, and functionality for capturing topographic data.

### 4.4. Potential Shortcomings in Capturing Accurate Topography

While 19 GCPs were placed throughout our study site (the area is relatively small at 160 m^2^), a survey design with more GCPs has potential to improve our results. This is because a shortcoming in SfM is vertical “doming” of the surface, which is a systematic error partially due to inaccurate correction of radial camera lens distortion (e.g., [9,24]). Topographic doming can be mitigated by the collection of more GCPs, but an accurate correction of the radial distortion is also required [9,42,43]. While we addressed photogrammetric issues of over-parameterization during camera self-calibration to include radial and tangential distortions, which has shown to decrease the doming effect [24], we used the JPEG-format image files pre-processed using onboard geometric adjustments by DJI. Lens distortion modelled in our DJI-based imagery photogrammetric workflow does not represent the true physical optics; instead, the residual distortions represent the generic lens geometry correction applied by DJI [42]. Therefore, the default DJI camera settings should be changed to include the raw (uncorrected) imagery for modeling and correcting systematic error due to lens distortion [42]. The choice of a self-calibrated camera calibration during bundle adjustment may still increase the doming effect in our results. Instead, a pre-calibrated camera model, such as derived by a common checkerboard routine, should be considered [44]. Another problem that may increase the systematic error is that the SfM algorithm estimates the internal camera parameters or camera pose a priori. An approach to manipulate the equations solved for SfM to eliminate all camera pose parameters has been presented [45]. However, the benefits of pose-free SfM still needs investigated before an agreed and easily implemented approach is employed to the community.

Doming is also caused by near parallel imaging directions chosen during flight planning [21,24,42,43]. We aimed to increase image geometry by choosing a −70-degree gimbal pitch angle with a double hatch pattern in the flight planning missions, which has been shown to reduce systematic error [46]. Another consideration that may increase systematic error is flight height. Systematic error has shown to be inversely proportional to flight height [9]. In this study, the pilots were restricted to fly below 30 m due to Federal Aviation Administration rules applied to the airspace, and thus it was not an option to fly above the 120 m threshold shown to reduce systematic error [9]. Therefore, alternative systematic-error correction methods (e.g., [43]) may help improve the results.

The art and science of capturing and processing accurate topographic data involves field survey requirements and rigorous photogrammetric workflows to compensate for the potential shortcomings in SfM-derived topographic data. In this study, we used current best practices when considering measurement precision (random error, e.g., standard deviation) in our analysis of the different sensors. However, a rigorous handling of measurement accuracy (systematic error or mean bias) is also necessary to correct for the systematic error so that these errors do not compromise precision-based changes in topography [42]. Future research should consider combining a rigorous precision (e.g., [23,24]) and accuracy (e.g., [42]) approach to collecting and processing accurate topographic data.

## 5. Conclusions

We brought together multiple sUAS systems simultaneously to assess their performance in a channelized stream with varying slopes and terrain roughness to help aid in future planning and budgeting of research where complex topographic change detection is critical. Five different commercially available sUAS from DJI were compared: (1) Matrice 600 Pro mounted with the DJI Ronin-MX gimbal caring a Sony a7iii camera, referred to as MaRS; (2) Inspire 2 carrying a Zenmuse X4S camera; (3) Phantom 4 Pro; (4) Mavic 2 Pro; and (5) Mavic Air Mini. A rigorous field and photogrammetric workflow must be replicated to reduce error and maintain consistency when comparing two or more datasets. Our workflow uses several proven data processing and analysis techniques by combining structure-from-motion to align images, Monte Carlo simulations to calculate spatially variable error, object-based image analysis to delineate topographic features, and MC32-PM algorithm to calculate statistically significant change detection for each topographic feature. This workflow can be used to monitor change in similar environments with complex topography.

Our results demonstrate that sUAS sensors with a rolling shutter speed, such as Mavic 2 Pro and Mavic Air Mini, should not be considered for monitoring environmental change. Instead, sUAS sensors with fine resolutions (≥20 MP), a wide range in global shutter speed, lens aperture, and ISO should be considered, such as MaRS, Inspire 2, and Phantom 4 Pro. Overall, the MaRS sensor achieved the lowest vertical median RMSE (0.06 m) and horizontal median RMSE (0.099 m) in complex topography and might be improved if the camera is pre-calibrated. It would also be a valuable direction for future research to assess, report, and incorporate error when comparing two or more datasets. Scientists and funding agencies seeking the best tradeoff between sensor quality, cost, portability, and functionality should consider a system such as Phantom 4 Pro. We hope that this study will stimulate the application of sUAS to topographic change detection and environmental monitoring.

## Figures and Tables

**Table 1:** Online Positioning User Service (OPUS) solution for the benchmark established in this study.

NAD83 2011 (EPOCH: 2010)	RMSE	State Plane Coordinates: SPC (3200 NC)
N 35°36’49.946”	0.002 (m)	Northing: 207,975.377 (m)
W 77°22’7.720”	0.006 (m)	Easting: 757,366.267 (m)
Ellipsoid height: −25.404 (m)	0.013 (m)	
Orthometric height:10.471 (m) [NAVD88 using GEOID 12B]	0.028 (m)	
Overall	0.015 (m)	

**Table 2:** Specifications of the five small unmanned aerial systems (sUAS) and their respective sensor used in this study.

Aircraft	Wind Resistance	Flight Time	Sensor	Lens	Aperture	Shutter Speed	ISO Range	Resolution
MaRS	18 mph	20 min	BSI CMOS	28 mm	f/2–f/22	Global: 1/8000–30 s	100–51,200	24 MP
Inspire 2	22 mph	27 min	1″ CMOS	24 mm	f/2.8–f/11	Global: 1/2000–8 s	100–12,800	20 MP
Phantom 4 Pro	22 mph	30 min	1″ CMOS	24 mm	f/2.8–f/11	Global: 1/2000–8 s	100–6400	20 MP
Mavic 2 Pro	18–24 mph	31 min	1″ CMOS	28 mm	f/2.8–f/11	Rolling: 1/8000–8 s	100–12,800	20 MP
Mavic Air Mini	18 mph	30 min	1/2.3″ CMOS	24 mm	f/2.8	Rolling: 1/8000–4 s	100–3200	12 MP

**Table 3:** Parameters for each flight using DJI GS Pro 3-Map area mission.

Parameter	MaRS	Inspire 2	Phantom 4 Pro	Mavic 2 Pro	Mavic Air
Shooting angle	Perpendicular to path	Perpendicular to path	Perpendicular to path	Perpendicular to path	Perpendicular to path
Capture mode	Hover and capture at a point	Equal distance interval	Hover and capture at a point	Equal distance interval	Equal distance interval
Capture interval	N/A	2.0 s	N/A	2.0 s	2.0 s
Flight course mode	Inside mode	Inside mode	Inside mode	Scan mode	Scan mode
Speed	3.5 m/s	12.4 m/s	3.5 m/s	2.8 m/s	3 m/s
Altitude (m)	23	23	23	23	23
Front overlap	80%	80%	80%	80%	80%
Side overlap	80%	80%	80%	80%	80%
Course angle	30° and 120°	30° and 120°	30° and 120°	30° and 120°	30° and 120°
margin	0	0	0	0	0
Gimbal pitch angle	−70°	−70°	−70°	−70°	−70°
Waypoints qty	167	38	35	52	56
Flight length (m)	909 m	953	787 m	1515 m	1693
Cover area	0.30 ha	0.54 ha	0.30 ha	0.56 ha	0.56 ha
GSD	0.5 cm/px	0.6 cm/px	0.6 cm/px	0.7 cm/px	0.9 cm/pix

**Table 4:** Camera models and their parameters tested in this study.

Camera Model	Parameters Included
Model B	focal length (*f*), principal point (*cx*, *cy*), radial distortion (*k*1, *k*2, *k*3)
Model C	focal length (*f*), principal point (*cx*, *cy*), radial distortion (*k*1, *k*2, *k*3), tangential distortion (*p*1, *p*2)

**Table 5:** Errors and camera models used when accessing ground control point (GCP) error and generating precision estimates.

Aircraft	Image Coordinates (pix)		GCP Ground RMSE (m)	Camera Model
	Reprojection RMSE	GCP Image RMSE		
MaRS	0.50	0.33	0.03	B
Inspire 2	0.79	0.30	0.03	B
Phantom 4 Pro	0.75	0.34	0.03	B
Mavic 2 Pro	0.82	1.49	0.03	B
Mavic Air Mini	1.95	0.45	0.03	C

## Data Availability

The data presented in this study are available on request from the corresponding author.
